# Sexual dysfunction after open abdominal aortic aneurysm repair: 16 years’ experience in a quaternary center and literature review

**DOI:** 10.1590/1677-5449.202301352

**Published:** 2024-02-05

**Authors:** Bruno Pagnin Schmid, Marcelo Vezzi Muce, Rodrigo Gonzalez Bocos, Fábio Hüsemann Menezes

**Affiliations:** 1 Universidade Estadual de Campinas - UNICAMP, Campinas, SP, Brasil.; 2 Hospital Israelita Albert Einstein - HIAE, São Paulo, SP, Brasil.

**Keywords:** aneurysm, erectile dysfunction, ejaculation, aneurisma, disfunção erétil, ejaculação

## Abstract

**Background:**

Open abdominal aortic aneurysm (AAA) repair can lead to sexual dysfunction (SD) in men.

**Objectives:**

To determine the prevalence of SD following open AAA repair, explore whether surgical techniques for aortic reconstruction can have a differential impact on the occurrence of SD, and summarize current knowledge in this field.

**Methods:**

Retrospective review of 100 patients submitted to open AAA repair between 1995 and 2010 in a quaternary center. Sexual dysfunction was assessed according to questions from the modified International Index of Erectile Function (IIEF), considering the condition before surgical repair and 3 months after surgery. The chi-square test, Fisher’s exact test, and Student’s *t* test were used for statistical analyses.

**Results:**

100 patients were included (mean age = 66.4 years old). Normal sexual activity, no sexual activity, erectile dysfunction, and retrograde ejaculation with preserved erectile function were found in 36%, 21%, 18%, and 24% of patients, respectively. The group of patients with no sexual activity was older (mean age = 72.3 years old *vs* 64.5 years old, p < 0.001). Erectile dysfunction prevalence was higher in patients submitted to an aorto-bifemoral bypass (p = 0.032). Retrograde ejaculation was more frequent in patients submitted to an aorto-aortic bypass (p = 0.007).

**Conclusions:**

Sexual function is a frequent condition intimately associated with the aortic reconstruction technique. The literature review found contradictory results regarding whether the endovascular approach is protective compared with open repair, but clearly demonstrated the importance of techniques targeting preservation of the internal iliac artery and the superior hypogastric plexus.

## Resumo

**Contexto:**

A correção aberta de aneurisma da aorta abdominal (AAA) pode causar disfunção sexual (DS) em homens.

**Objetivos:**

Determinar a prevalência de DS após correção aberta de AAA, determinar se a técnica de reconstrução aórtica pode estar correlacionada com o surgimento de DS e resumir os achados mais relevantes da literatura relacionados a esse tema.

**Métodos:**

Estudo retrospectivo de todos os pacientes submetidos a correção aberta de AAA entre 1995 e 2010 num centro quaternário. A DS foi avaliada por meio de questões baseadas no escore *International Index of Erection Function* no pré-operatório e 3 meses após a cirurgia. A análise estatística foi realizada com os testes do qui-quadrado, exato de Fisher e *t* de Student.

**Resultados:**

Cem pacientes foram incluídos (idade média = 66.4 anos). Atividade sexual normal, atividade sexual ausente, disfunção erétil (DE) e ejaculação retrógrada (ER) com função erétil preservada foram observadas em 36%, 21%, 18% e 24% dos pacientes, respectivamente. O grupo de pacientes com atividade sexual ausente foi significativamente mais velho (idade média = 72.3 anos vs. 64.5 anos, p < 0.001). A DE foi mais prevalente em pacientes submetidos a enxerto aorto-bifemoral (p = 0.032), enquanto a ER foi mais prevalente em pacientes submetidos a enxerto aorto-aórtico (p = 0.007).

**Conclusões:**

A DS é uma condição frequente e intimamente associada à reconstrução aórtica empregada. A literatura apresenta resultados contraditórios sobre qual correção de AAA (aberta ou endovascular) resulta em mais DS, mas demonstra a importância de técnicas com preservação das artérias hipogástricas e do plexo hipogástrico superior.

## INTRODUCTION

Abdominal aortic aneurysm (AAA) is a prevalent condition in the elderly population.^1-3^ More than 30,000 patients undergo elective repair each year in the USA.^4^ With the progressive increase in life expectancy, the prevalence of AAA is also growing.^3^ Surgical repair is still the only definitive treatment option for AAA and sexual dysfunction (SD), defined as persistent or recurrent disorders of sexual desire/interest, arousal, or orgasm or sexual pain, is a poorly described complication of surgical repair.^1,2,5^

Retrograde ejaculation (RE) and erectile dysfunction (ED) are two clinical presentations of SD that have a decisive impact on the quality of life of patients treated for AAA with open repair (OR).^6-9^ Retrograde ejaculation is defined as failure of closure of the bladder neck resulting in reflux of semen into the bladder.^10^ Erectile dysfunction is defined as the inability to achieve and/or maintain sufficient penile erection for satisfactory sexual performance.^11^ Some authors have tried to establish a correlation between the surgical AAA repair technique and the impact on the occurrence of SD, but with contradictory results.^3,4,6-9,12^

Against this background, the aim of this study is to determine the prevalence of SD following open AAA repair and explore whether surgical techniques used for aortic reconstruction can have a differential impact on the occurrence of SD. An additional objective of this research is to summarize current knowledge on the occurrence of SD following surgical AAA repair.

## METHODS

**Figure 1:**
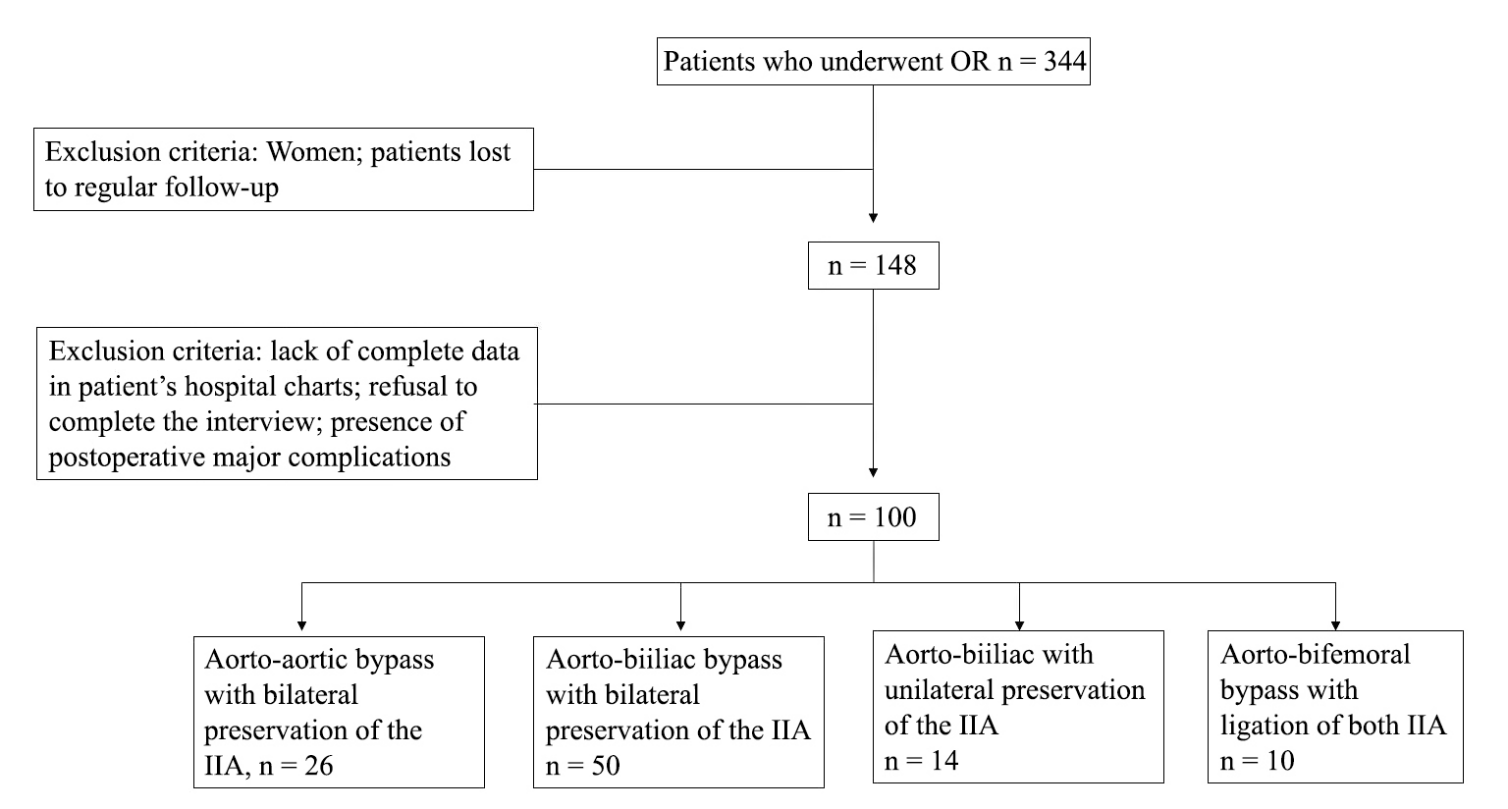
Flow chart of patients included in the study. IIA = internal iliac artery; OR = open repair.

This is a retrospective cohort of patients who underwent OR in a public university hospital over a 16-year period from 1995 to 2010. Woman and patients lost to regular follow up were excluded, resulting in 148 individuals. From this group, 48 patients were eliminated due to lack of complete hospital chart data, refusal to complete the interview, or presence of major postoperative complications, such as myocardial infarction, stroke, permanent dialysis, and mesenteric ischemia. Patients who already had SD or no sexual activity prior to the surgical procedure were also excluded from the final analyses. 100 patients were personally questioned about SD and had complete data concerning sexual function and thus constituted the study group (Figure 1). Interviews were conducted during regular follow-up appointments. This study was approved by the Institutional Ethics Committee (CAAE 0984.0.146.000-11, decision number 1085/2011).

Sexual function was assessed according to questions from the modified International Index of Erectile Function (IIEF), a highly regarded tool for assessing SD.^13^ Since the original IIEF is not well-suited to use as a simple screening measure and there are no objective diagnostic tests available to physicians for confirmation of SD during medical appointments, we used an abridged questionnaire based on the IIEF during regular follow-up.^13,14^ All patients were asked to answer the questionnaire considering the condition before surgical repair and 3 months after surgery. The 3-month period is commonly used to assess SD after prostatectomy and, because of this, was also used in this study.^15^Table 1 illustrates the questions used. Responses were not scored and no cutoff limit was established. All patients received the clinical diagnosis of SD after a multidisciplinary team of vascular surgeons and urologists met to discuss their answers, patient histories, and physical examination findings.

**Table 1:** Questions from the International Index of Erectile Function.

Questions from the International Index of Erectile Function
1. How often were you able to get an erection during sexual activity?
2. When you attempted sexual intercourse, how often were you able to penetrate (enter) your partner?
3. During sexual intercourse, how often were you able to maintain your erection after you had penetrated (entered) your partner?
4. How much have you enjoyed sexual intercourse?
5. When you had sexual stimulation or intercourse, how often did you ejaculate?
6. How satisfied have you been with your overall sex life?

The consultations and results were annotated in the patients’ charts and in an electronic database (Microsoft Access®) to create a retrospective review registry. A descriptive analysis of the patients’ demographics was performed. The chi-square test, Fisher’s exact test, and Student’s *t* test were used for statistical analyses. The level of significance was set at p < 0.05. The investigator who performed the statistical analyses was blinded to group allocation to minimize bias. To describe the prevalence of SD following open AAA repair, with tolerable error of ±5% and confidence interval of 95%, the study sample was calculated based on the results obtained by Gallitto et al.,^16^ who estimated a proportion of 31% in a study with 115 patients.

## RESULTS

### Patients’ demographics

There was a predominance of Caucasian patients in the seventh decade of life (mean age = 66.4 years, range = 48.8 to 85.7). Systemic hypertension was present in 99% of the patients, a history of smoking in 92%, dyslipidemia in 33%, and diabetes in 14%. Mean follow-up time was 6.25 years.

### Surgical techniques

**Figure 2:**
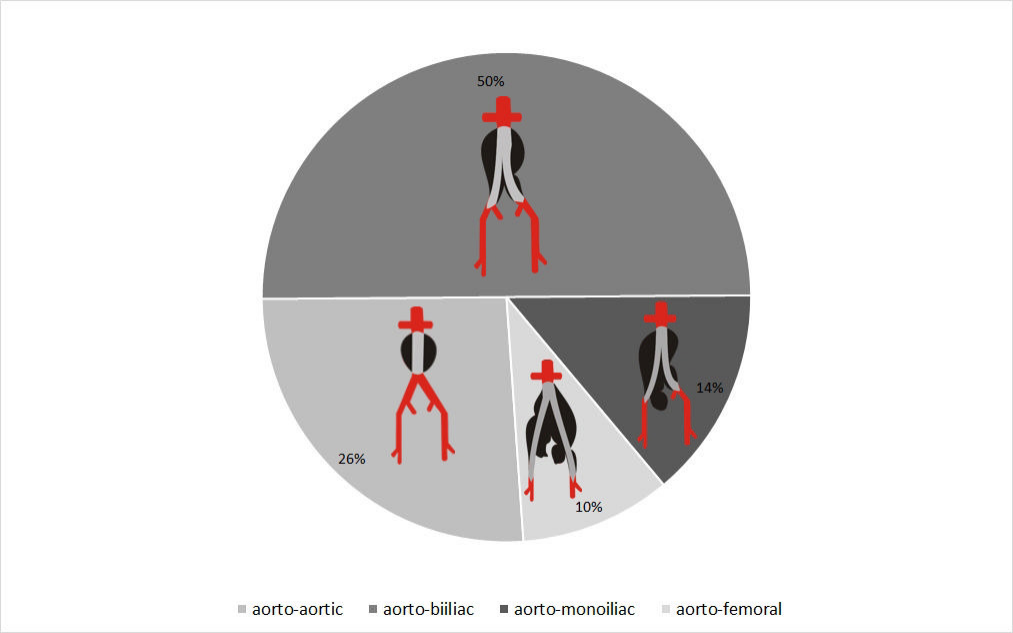
Distribution of patients according to surgical reconstruction techniques.

Open repair techniques included aorto-aortic bypass with bilateral preservation of the internal iliac artery (IIA), aorto-biiliac bypass with bilateral IIA preservation, aorto-biiliac with unilateral IIA preservation, and aorto-bifemoral bypass with ligation of both IIAs. The distribution of these operations is presented in Figure 2.

### Sexual dysfunction

The correlations between type of aortic reconstruction and sexual dysfunctions were analyzed for 100 patients and the data summarized in Table 2. ED was significantly more frequent in aorto-bifemoral bypass patients (p< 0.01). Aorto-aortic, aorto-biiliac, and aorto-monoiliac configurations were not statistically different regarding this endpoint (p = 0.111, p = 0.581, and p = 0.205, respectively). RE was significantly more frequent in aorto-aortic bypass patients (p = 0.007). Aorto-bifemoral, aorto-biiliac, and aorto-monoiliac configurations were not statistically different regarding this endpoint (p = 0.274, p = 0.337, p = 0.111, respectively).

**Table 2:** Sexual dysfunction according to aortic reconstruction technique.

	No sexual activity (Total = 21)	Normal activity (Total = 34)	ED (Total = 18)	RE (Total = 27)
Aorto-aortic (Total=26)/ percentage of group	4/ 15.4%	7/ 30%	2/ 7.7%	13/ 50%
Aorto-biiliac (Total=50)/ percentage of group	12/ 24%	21/ 42%	6/ 12%	11/ 22%
Aorto-monoiliac (Total=14)/ percentage of group	2/ 14.3%	6/ 42.86%	5/ 35.71%	1/ 7.14%
Aorto-bifemoral (Total=10)/ percentage of group	3/ 30%	0/ 0%	5/ 50%	2/ 20%

ED = erectile dysfunction, RE = retrograde ejaculation.

**Figure 3:**
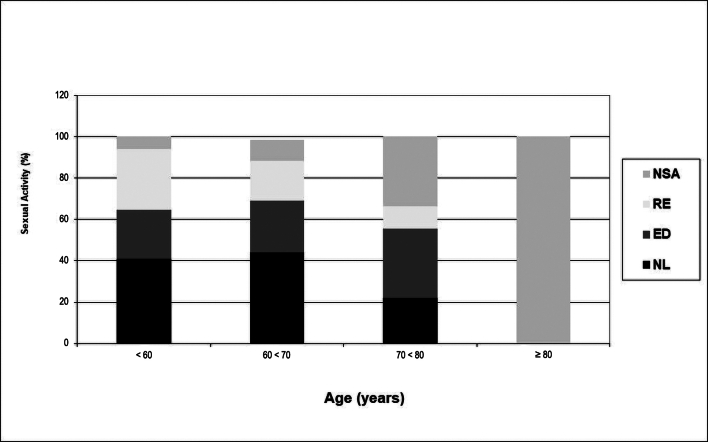
Sexual function according to age in patients who underwent open AAA repair. NSA = no sexual activity; RE = retrograde ejaculation; ED = erectile dysfunction; NL = normal sexual activity.

The group that reported no sexual activity was significantly older than the other groups (mean age = 72.3 years old *vs.* 64.5 years old, p<0.001), as illustrated in Table 3 and Figure 3.

**Table 3:** Mean age of different groups.

**Sexual status**	**Age in years (mean)**	**No sexual activity vs. other groups (mean age/number of patients)**	p-value*
No activity (Total = 21)	72.3	72.3 years (21 patients)	< 0.001
Normal activity (Total = 34)	63.9	64.5 years (79 patients)	
ED (Total = 18)	63.7		
RE (Total = 27)	65.8		

ED = erectile dysfunction; RE = retrograde ejaculation.

* Student’s *t* test.

No differences were observed in relation to systemic arterial hypertension (p = 0.406), diabetes (p = 0.435), smoking history (p = 0.800), or dyslipidemia (p = 0.216).

## DISCUSSION

A literature search of the MEDLINE database was performed in May 2020 to identify original publications and literature reviews from the last 5 years pertaining to retrograde ejaculation and erectile dysfunction after OR. Search strategies included the following Medical Subject Headings terms = “aortic aneurysm, abdominal”, “sexual dysfunction, physiological”, “erectile dysfunction”, and “ejaculation”. No language restrictions were applied. A total of 14 studies were found in the MEDLINE database. Two studies were excluded after abstract screening and two other studies were excluded after full-text screening because they didn’t contribute any specific data concerning SD assessment following AAA surgical repair. Ten studies were included in the final analysis and their main results are summarized in Table 4.

**Table 4:** Sexual dysfunction according to different reports.

**Author**	**Study Design**	**Number of patients/studies**	**Main results**
Kalteis et al.^17^	Retrospective	106 (82 EVAR vs. 24 EVAR with IIA embolization)	EVAR with IIA embolization was associated with new onset ED (17.3% vs. 42.9%; p = 0.043).
	Single-center		
	EVAR vs. EVAR with IIA embolization		
Majd et al.^18^	Prospective	100 (70 EVAR vs 30 OR)	Increase of ED in both groups (53.3.% OR vs. 58.8% EVAR, p = 0.412).
	Single-center		
	OR vs. EVAR		
Machado et al.^19^	Retrospective	171	ED was more frequent in the elderly group (p<0.05)
	Single-center		
	EVAR (patients were divided according to age groups < 70, 70-80, and > 80 years)		
Shin et al.^20^	Retrospective	14	No cases of ED
	Single-center		
	EVAR (bifurcated-bifurcated repair)		
Bosanquet et al.^21^	Meta-analysis	2671	Higher rates of ED after coiling (11.6% Coils vs. 3.03% Plugs)
	EVAR (coils vs. plugs)		
Regnier et al.^22^	Meta-analysis	29 studies	Post-operative ED prevalence= (OR 7.4 to 79% vs. EVAR 4.7 to 82%). Post-operative RE prevalence= (OR 3.3 to 9% vs. Laparoscopic repair 6 to 6.6%)
Kudo et al.^23^	Narrative Review	NR	Emphasized the importance of preservation of blood flow from IIA and of adequate preservation of the superior hypogastric plexus and lumbar splanchnic nerves
Gallitto et al.^16^	Retrospective	115 (58 EVAR, 57 OR)	RE occurred more often in OR patients (31%) than in EVAR patients (2%) (*P* = 0.001).
	Single-center		
	EVAR vs. OR		
van Schaik et al.^24^	A questionnaire-based study aiming to analyze care for sexual health by medical specialists	101	A gap exists in knowledge of pathophysiology and anatomy. Vascular surgeons lack sexual counseling skills
Dariane et al.^25^	Prospective. Multicenter.	25 (8 EVAR/ 13 Laparoscopic repair)	RE is frequent in both groups (Laparoscopic=61.5%/ EVAR=12.5%) Laparoscopic AAA repair caused no onset of ED or SD
	EVAR vs. Laparoscopic repair		
Schmid et al. (This study)	Retrospective	100	The aorto-bifemoral configuration was the technique most associated with ED (p=0.03) and the aorto-aortic configuration was the technique most associated with RE (p= 0.01). OR is associated with 64% of SD, 24% of RE, 18% of ED, and 21% of no sexual activity
	Single-center		
	OR (aorto-bifemoral bypass vs. Aorto-aortic vs. aorto-biiliac vs. aorto-monoiliac)		

ED = erectile dysfunction; EVAR = endovascular aneurysm repair; IIA = internal iliac artery; NR = not reported; OR = open repair; RE = retrograde ejaculation; SD = sexual dysfunction.

The erection is essentially a vasomotor phenomenon controlled by IIA perfusion of the cavernous body and parasympathetic fibers located in the pelvis. Presence of advanced aneurysmal disease with a need for ligation of the IIA during an aorto-bifemoral bypass could explain the higher prevalence of potency loss in this type of aortic reconstruction. The statistically higher prevalence of ED after aorto-bifemoral bypass observed in this study corroborates this hypothesis.

In parallel, RE is caused by sympathetic fibers located anterior to the common iliac arteries that are often injured during aorto-aortic bypass, because of common iliac dissection and clamping and placing the suture line at the aortic bifurcation, which explains why this type of surgery is associated with higher rates of RE.^5,26-29^

Kudo et al.^23^ emphasized the importance for prevention of ED of preserving the blood flow from at least one IIA and the importance for prevention of RE of adequate preservation of the superior hypogastric plexus and lumbar splanchnic nerves.

van Schaik et al.^24^ conducted a study to evaluate vascular surgeons’ knowledge about these basic principles of SD after open AAA repair and possible nerve-preserving techniques in a Dutch population and, surprisingly, found that there was a gap in their knowledge of pathophysiology and anatomy concepts, highlighting the need for more education regarding sexual counseling during vascular surgical training.

Progressive sexual deterioration according to age is a well-established phenomenon and was also reaffirmed in the present study, since the group of patients with no sexual activity was significantly older. In this study, diabetes had no influence on sexual function, probably because of the low prevalence of diabetes in the cohort. Machado et al.^19^ performed a retrospective review and also identified age as a contributing factor to SD after EVAR.

Our findings are in agreement with the existing literature, confirming the high prevalence of SD after OR.^16,19,22^ However, after performing the literature review, we did not find any studies that explored whether the choice of open surgical techniques in aortic reconstruction can have a differential impact on the occurrence of SD, highlighting the importance of the present paper. There are some confounding factors regarding onset of SD besides the surgical procedure itself, such as cardiovascular diseases, absence of a sexual partner, and low libido, which were not individually analyzed in each group of patients according to the type of aortic reconstruction. This constitutes a limitation of this study.

In a meticulous literature review, Regnier et al.^22^ could not find a clear conclusion on whether the endovascular approach is protective compared with open repair, since results are contradictory. In a prospective series, Majd et al.^18^ found increased ED after AAA repair, but the difference between open repair and endovascular repair was not statistically significant. Gallitto et al.^16^ found a higher prevalence of RE in patients submitted to OR than in patients submitted to EVAR. In a single-center prospective study, Dariane et al.^25^ found interesting results regarding laparoscopic AAA repair. In the population studied, this particular technique caused no onset of erectile or sexual dysfunction.

Analyzing studies that just enrolled patients submitted to EVAR, Bosanquet et al.^21^ performed a meta-analysis including 61 non-randomized studies and 2671 patients. The authors observed an ED prevalence of 10.2% of the male patients, with higher rates after coiling than after plugs. They therefore suggested that plugs could be considered preferential to coils, placed as proximally in the IIA as possible. Kalteis et al.^17^ confirmed an increase in SD when embolization and coverage of the hypogastric was performed during EVAR. In a small retrospective review, Shin et al.^20^ reported using bifurcated-bifurcated endovascular repair of aortoiliac aneurysms, preserving perfusion to the IIA, with promising results demonstrating no SD after surgical repair in 14 patients.

Limitations of this study include the limited sample size, single-center design, and retrospective analyses. However, the incidence of SD following open AAA repair was determined and the correlations between type of SD and aortic reconstruction technique were explored.

## CONCLUSION

There is a high incidence of SD following open AAA repair. Preservation of the internal iliac artery and the sympathetic preaortic nerves are of the uttermost importance to the impact on sexual performance and must be targeted during open AAA repair. The aorto-bifemoral configuration was the technique most associated with ED and the aorto-aortic configuration was most associated with RE. Age was correlated with a lack of sexual activity. Since sexual performance plays a key role in quality of life, this study highlights the importance of informing patients of this potential side effect before surgery and of choosing the most appropriate surgical technique. The literature review found contradictory results on whether the endovascular approach is protective compared with open repair, but clearly demonstrated the importance of techniques targeting preservation of the IIA and the superior hypogastric plexus. It also summarizes the real need to inform patients undergoing AAA surgical repair about this potential side effect.
